# Improving Last-mile Delivery of COVID-19 Vaccines: The Cluster Strategy

**DOI:** 10.3126/nje.v11i3.39544

**Published:** 2021-09-30

**Authors:** Anila Varghese, Surachna, Sidharth Sekhar Mishra

**Affiliations:** 1 Anila Varghese, Junior Resident, Department of Community Medicine, King George’s Medical University, Lucknow, India; 2 Indepenedent Researcher, India; 3 Assistant Professor, International Institute Of Health Management Research, Delhi, India

##  

Sir,

Since the first case of SARS-COV-2 infection in December 2019, COVID-19 has claimed more than forty-three lakh lives worldwide [1]. As of August 2021, India has had 3.2 crore cases and 4.3 lakh deaths [2]. During the second wave of COVID-19, the country witnessed an acceleration in the number of cases, reduced supplies of essential treatments, and increased deaths particularly in the young population [3].

Vaccination against the virus is the most effective way to prevent infection and save lives. India began the COVID-19 vaccination on 16th January 2021, for health care and front-line workers, extending to those above sixty years and those above 45 years with co-morbidities from March 2021 and all citizens over 18 years from May 1, 2021. Since March 2021, the private sector has also joined in its vaccination drive to speed up the process [4]. India’s vaccination programme currently includes two vaccines – Covishield, the Oxford-Astra Zeneca vaccine manufactured by the Serum Institute of India and Covaxin by Bharat Biotech [5]. Four other vaccines, Sputnik V, Moderna, Janssen and Zydus Cadilla COVID-19 vaccines, have also received Drug Controller General of India (DCGI) approval for restricted use in emergencies [6].

Over five hundred million vaccine doses have been administered in the country (2). As of August 19, 2021, around 13% of the country’s adult population are fully vaccinated while over 43% had received at least one dose of COVID-19 vaccines [7,8]. Yet, around 94 crore beneficiaries over 18 years of age are eligible for the vaccination [9]. With a population of 1360 million, India has a long way to go to ensure adequate vaccination coverage and attain herd immunity.

It is estimated that 285 million doses per month will be needed over the next five months to vaccinate all remaining adults by the end of 2021[10]. Many states have faced a shortage of COVID-19 vaccines [11–13]. Yet, on 16th August 2021, the central government has assured that more than 56.81 crore vaccine doses have been provided to the states/union territories (UTs) and more than 2.89 crore balance and unutilised doses are still available with states/UTs and private hospitals [14]. Once the issue of supply is solved, accessibility and acceptability are the main barriers to improving vaccination coverage.

All states have developed strategies for increasing COVID-19 vaccination coverage. Uttar Pradesh (U.P), the most populated state, has administered over 57 million doses of the COVID-19 vaccine [2]. To accelerate the vaccination coverage for its 200 million population, the state has planned a cluster approach to vaccination in rural areas [15].

The cluster strategy is a micro-plan for vaccination by the government of U.P involving intensive mobilisation activities followed by vaccination at centres set up in schools, Panchayat Bhavans and other selected places [16]. Beginning in rural areas, in the pilot phase, one-third of the developmental blocks in all districts were divided into four clusters of 10-12 villages. Three days of mobilisation activities were being conducted by the public awareness team involving the village heads (Gram Pradhans), Accredited Social Health Activists (ASHAs), Anganwadi workers (AWWs) and school teachers. The beneficiaries (all persons 18 years and above) were identified and notified of the venue and time for vaccination. With the help of this cluster strategy, the state government achieved the target to inoculate 10 million people in June [17]. This article explores the advantages and challenges of this strategy of vaccination and proposes measures to improve the utility of the cluster approach to increase last-mile vaccine acceptance and accessibility.

### Benefits of the Cluster strategy

1. Community involvement: Mobilisation activities
  At the beginning of the vaccination drive, resistance in rural areas of U.P was high with many unfortunate incidents like villagers jumping into a river to escape vaccination [18]. This leads us to consider the concept of vaccine acceptance which represents a spectrum of behaviours and beliefs from the rejection of all vaccines to active support of immunization recommendations [19]. It will depend on many factors including availability, accessibility, hesitancy, social and behavioural factors including cultural support, religious, educational or philosophical views. Providing scientifically sound advice in a socially acceptable manner, clarification of doubts, dispelling myths and installing confidence are components of a good public awareness campaign. The fact that these mobilisers are residents of the same area will help in improving confidence. A people-centred and comprehensive approach, modelled on listening to the intended beneficiaries and stakeholders is vital. This can be achieved through the mobilisation component of the cluster strategy.
2. Improving accessibility with a focus on last-mile delivery.
  Accessibility is an important factor determining vaccine uptake. COVID-19 vaccination is provided free of cost at public health facilities but hesitancy arises if these vaccination centres are difficult to reach. Booking slots on the CoWin portal has also been difficult for many beneficiaries. Providing vaccines at a centre near homes like the subcentres under the public health system, schools, Anganwadi centres and makeshift sites can decrease the hurdles of accessibility. This will reduce expenses related to travel and loss of wages for those missing work while travelling far to get vaccinated. The cluster approach is also along the lines of the Near to Home COVID Vaccination Centres (NHCVC) for Elderly and Differently Abled Citizens [20].

A gender gap has been observed in the vaccination statistics which show that women constitute 46.8% of the beneficiaries to date [21]. With due consideration to the social and cultural practices in many areas, it is difficult for them to travel long distances to get vaccinated, especially unaccompanied. This, coupled with practical difficulties like leaving young children behind and household chores will reduce vaccine uptake. Bringing vaccines closer to homes will play an important role in reducing the gender divide.

### Challenges

Only 0.006% of cases of Adverse Events Following Immunisation (AEFI) have been reported among all the vaccinated in the country (21). Yet, the main fear of the health workers regarding the cluster approach is concerning adverse events and their management at the peripheral level. Some solutions for this are ensuring the presence of a medical officer, arrangement of anaphylaxis kit, having an ambulance on stand-by and training of health workers on basic resuscitative measures. After all, even before the COVID-19 vaccination, under the Universal Immunisation Programme (UIP), vaccination services for children and pregnant women have been provided at subcentres and Anganwadi centres. Auxiliary Nurse Midwives (ANMs) have been trained about AEFI during these sessions. Through UIP, the government is currently, immunizing 26 million children and 30 million pregnant women annually [22]. These facts can help dispel fears about AEFI and its management.

It must also be emphasized that this strategy is only practically possible if an adequate supply of vaccines is available. This can be ensured by the central and respective state governments.

A SWOT analysis of the cluster approach reveals that the strengths and opportunities provided by this strategy outweigh the challenges of AEFI and vaccine shortage (Figure 1).

## Conclusion

The cluster approach to vaccination ensures accessibility, includes community participation and is provided free of cost. This is thus in line with the concept of Primary Health Care (PHC) which is essential health care made universally accessible to individuals and families in the community by means acceptable to them, through their full participation and at a cost the community and country can afford. Popularising this strategy and applying it to other states in a tailored manner based on social and cultural practices can give the nation the necessary momentum to attain the target of herd immunity quickly and curb the COVID-19 pandemic.

Dr. Anila Varghese, Junior Resident, Department of Community Medicine, King George’s Medical University, Lucknow, India

Dr. Surachna, Indepenedent Researcher, India.

Dr. Sidharth Sekhar Mishra, Assistant Professor, International Institute Of Health Management Research, Delhi, India

Dated the 02 September 2021

## Figures and Tables

**Figure 1:**
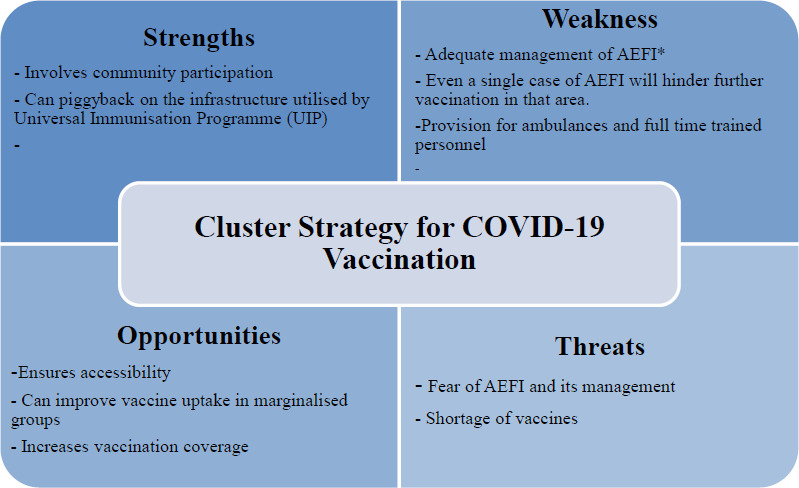
SWOT Analysis of Cluster Strategy for COVID-19 Vaccination *AEFI= Adverse events following Immunisation
